# Supplementary material for: Manipulation of time-dependent multicolour evolution of X-ray excited afterglow in lanthanide-doped fluoride nanoparticles
Source: Nat Commun. 2022 Sep 30;13:5739. doi: 10.1038/s41467-022-33489-1 (PMC9525643; doi:10.1038/s41467-022-33489-1)
Supplement: Supplementary file 1 — Supplementary Information [file 41467_2022_33489_MOESM1_ESM.pdf]

Supplementary Information

Manipulation of time-dependent multicolor evolution of X-ray excited afterglow in lanthanide-doped fluoride nanoparticles

Lei Lei[1], Yubin Wang[1] , Weixin Xu[1], Renguang Ye[1], Youjie Hua[1], Degang Deng[1], Liang Chen[1], Paras N. Prasad[2], Shiqing Xu[1]

[1]Key Laboratory of Rare Earth Optoelectronic Materials and Devices of Zhejiang Province, Institute of Optoelectronic Materials and Devices, China Jiliang University, Hangzhou 310018, People's Republic of China

[2] Institute for Lasers, Photonics, and Biophotonics and Department of Chemistry, University at Buffalo, State University of New York, Buffalo, New York 14260, United States

Correspondence and requests for materials should be addressed to P.N. (email: pnprasad@buffalo.edu) or to X.S. (email: shiqingxu@cjlu.edu.cn)

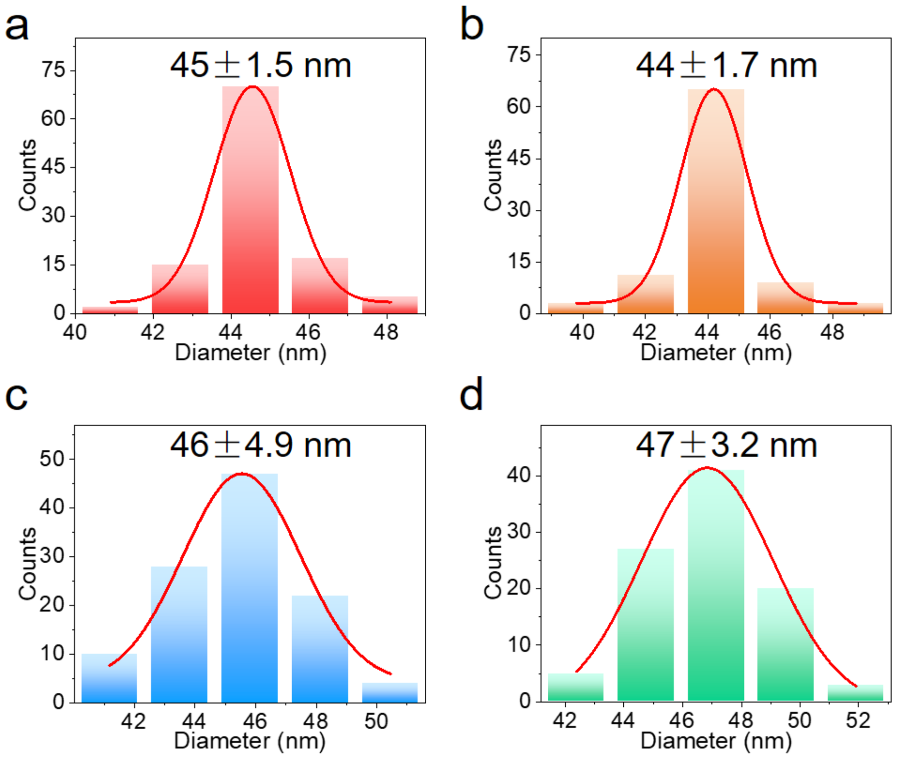

Supplementary Fig. 1 Histograms of size distributions of the $NaLuF_4$: Gd/Tb NPs prepared with different [Na]/[RE], 2.5 **(a)**, 5 **(b)**, 7.5 **(c)** and 10 **(d)**. Source data are provided as a Source Data file.

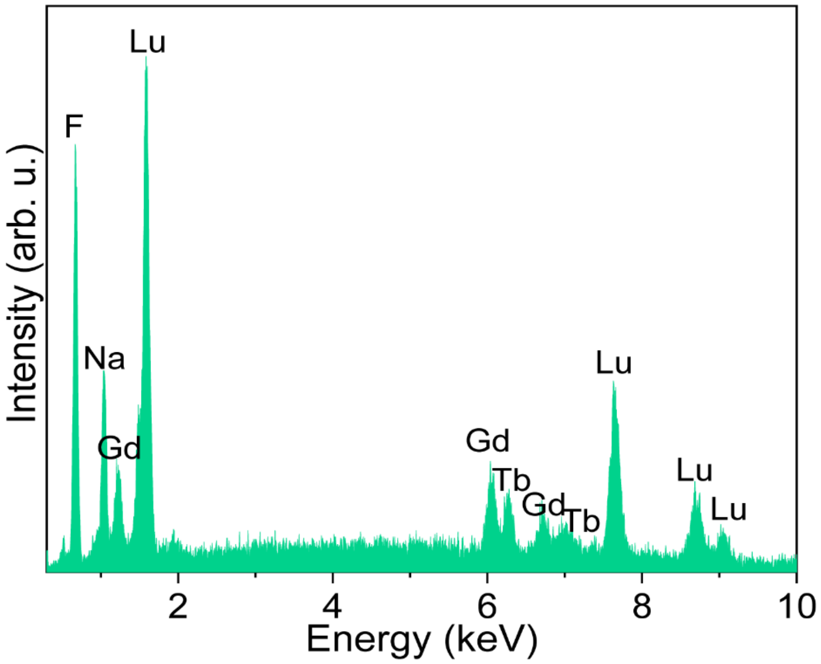

Supplementary Fig. 2 EDS spectrum of the $NaLuF_4$: Gd/Tb NPs prepared with [Na]/[RE] of 10. Source data are provided as a Source Data file.

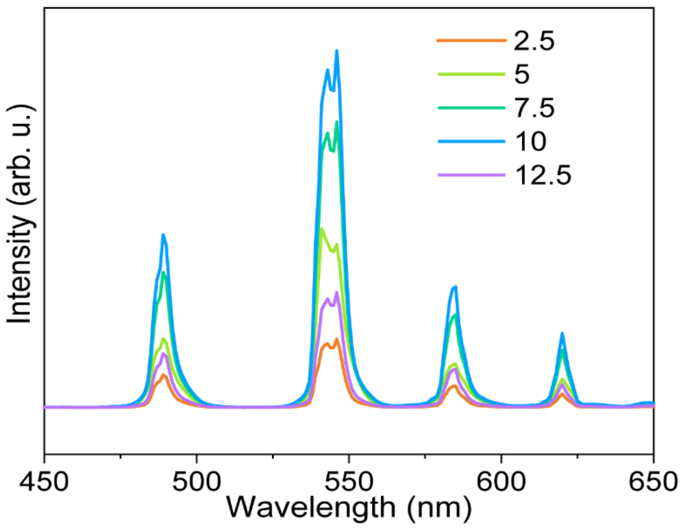

Supplementary Fig. 3 X-ray excited prompt luminescence (XEOL) of the $NaLuF_4$: Gd/Tb NPs prepared with different [Na]/[RE] (2.5, 5, 7.5 and 10, 12.5). Source data are provided as a Source Data file.

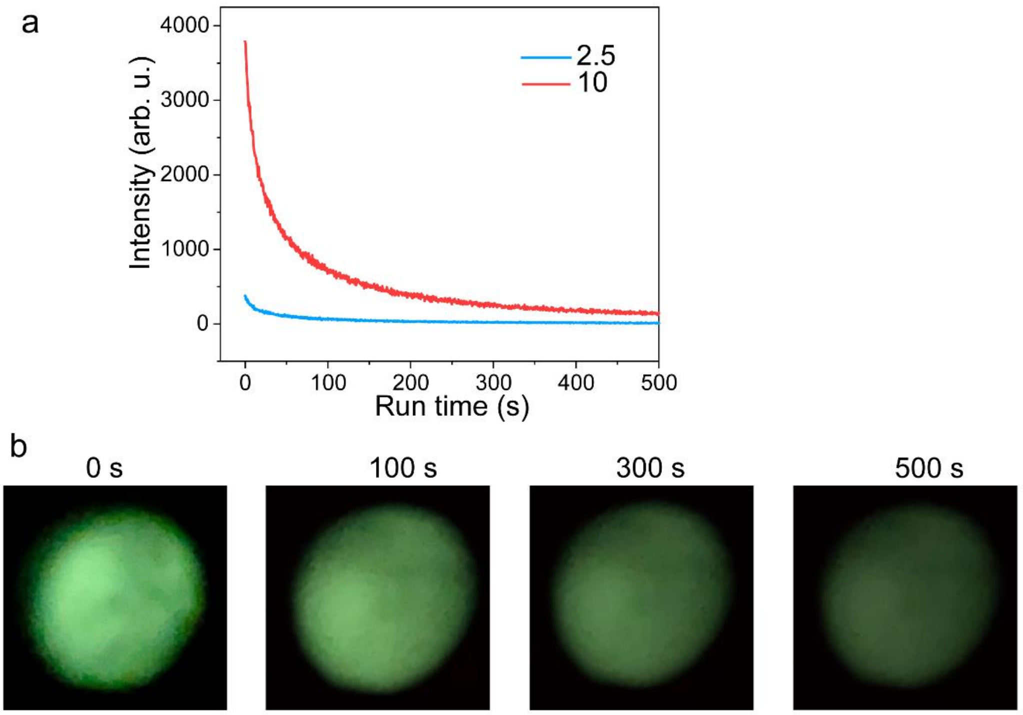

Supplementary Fig. 4 a XEA decay curves of the $NaLuF_4$: 15Gd/ 15Tb NPs prepared with [Na]/[RE] of 2.5 and 10. **b** Photographs of the $NaLuF_4$: 15Gd/ 15Tb NPs prepared with [Na]/[RE] of 10 at different time after the caseation of X-rays (30 kV for 5 min). Source data are provided as a Source Data file.

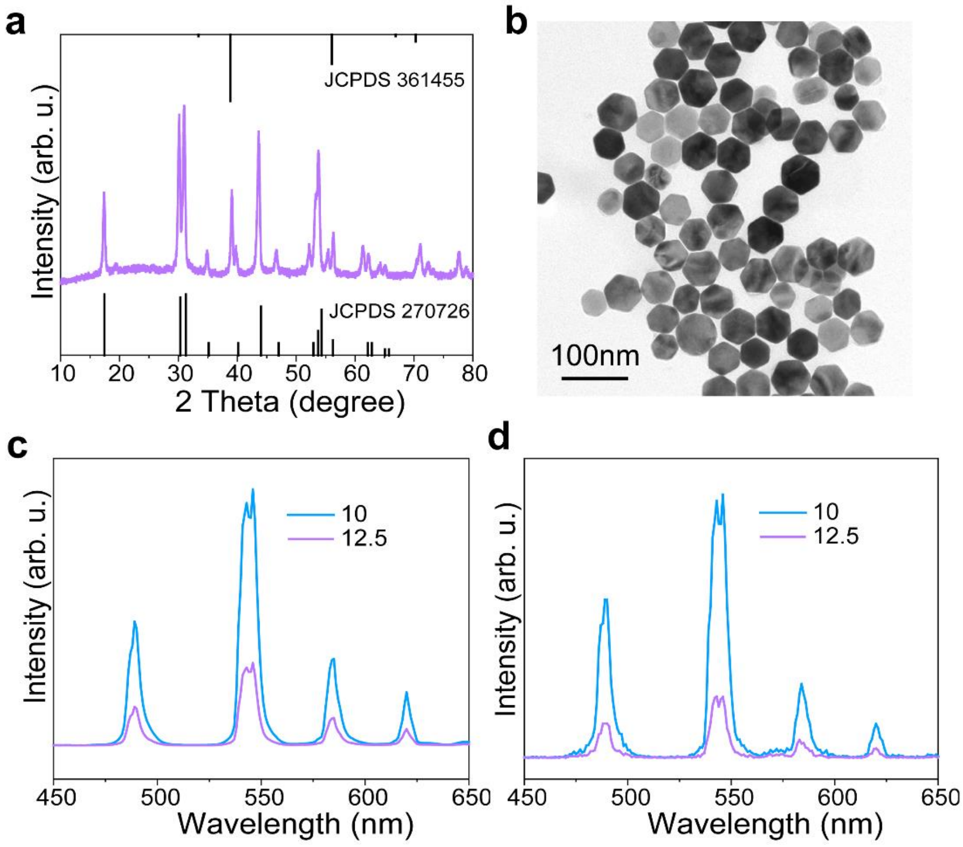

Supplementary Fig. 5 XRD pattern **(a)**, TEM image **(b)** of the $NaLuF_4$: Gd/Tb NPs prepared with [Na]/[RE] of 12.5. XEOL **(c)** and XEA **(d)** of the $NaLuF_4$: Gd/Tb NPs prepared with [Na]/[RE] of 10 and 12.5. The JCPDS 361455 and 270726 represent the standard data of cubic NaF and hexagonal $NaLuF_4$. Source data are provided as a Source Data file.

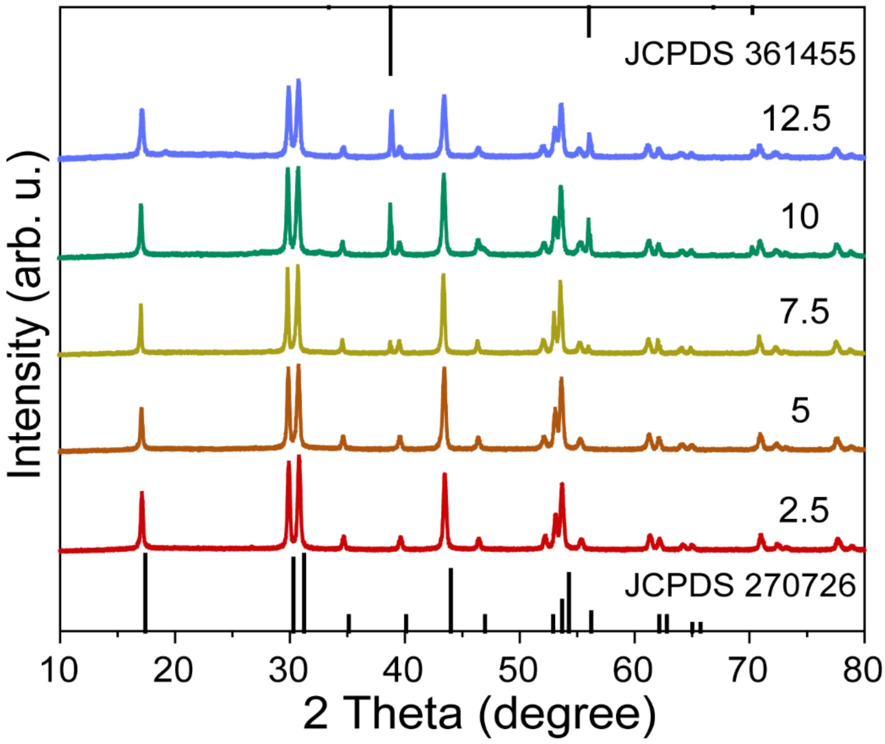

Supplementary Fig. 6 XRD patterns of the $NaLuF_4$: Gd/Tb NPs prepared with different [Na]/[RE] (2.5, 5, 7.5, 10 and 12.5) and [F]/[RE] of 5. Source data are provided as a Source Data file.

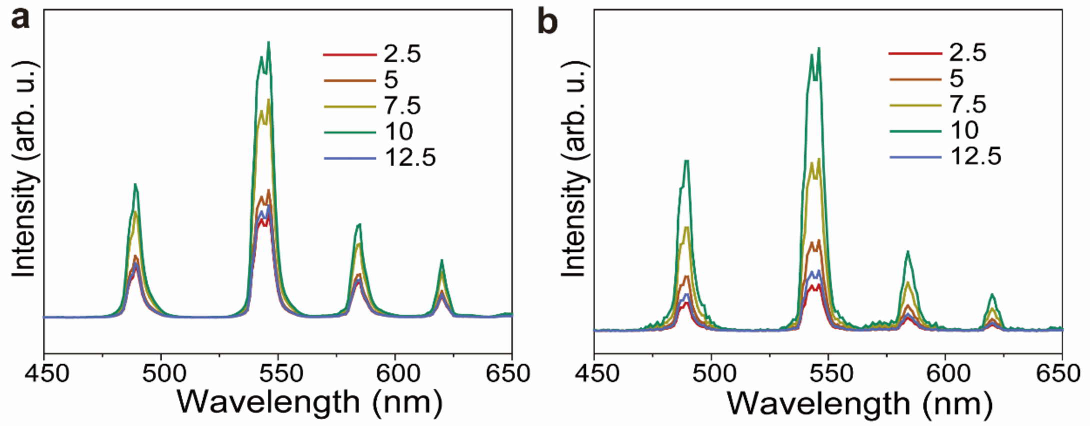

Supplementary Fig. 7 XEOL (**a**) and XEA (**b**) spectra of the $NaLuF_4$: Gd/Tb NPs prepared with different [Na]/[RE] (2.5, 5, 7.5, 10 and 12.5) and [F]/[RE] of 5. Source data are provided as a Source Data file.

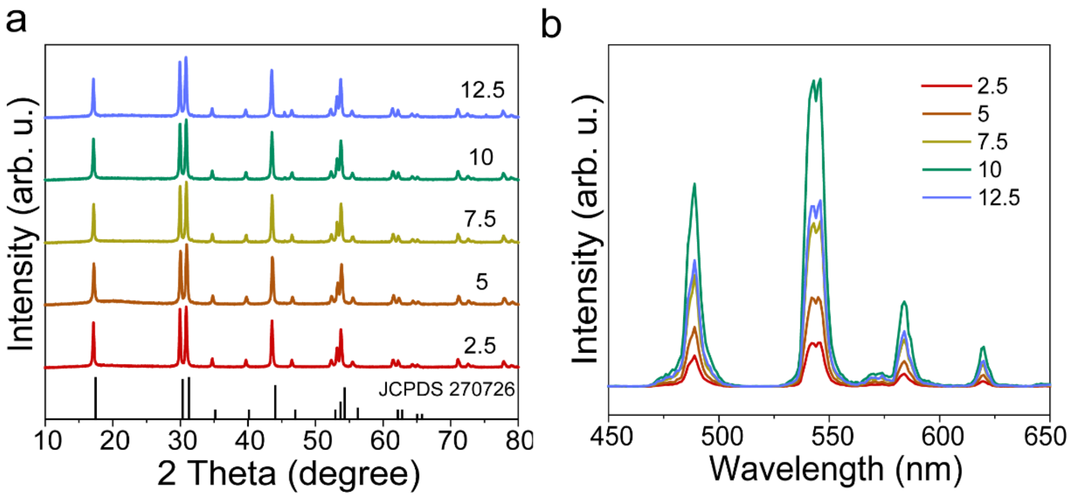

Supplementary Fig. 8 XRD patterns (**a**) and XEA spectra (**b**) of the $NaLuF_4$: Gd/Tb NPs ([Na]/[RE] = (2.5, 5, 7.5, 10, 12.5 and [F]/[RE] = 5) after washing with water-ethanol mixture. Source data are provided as a Source Data file.

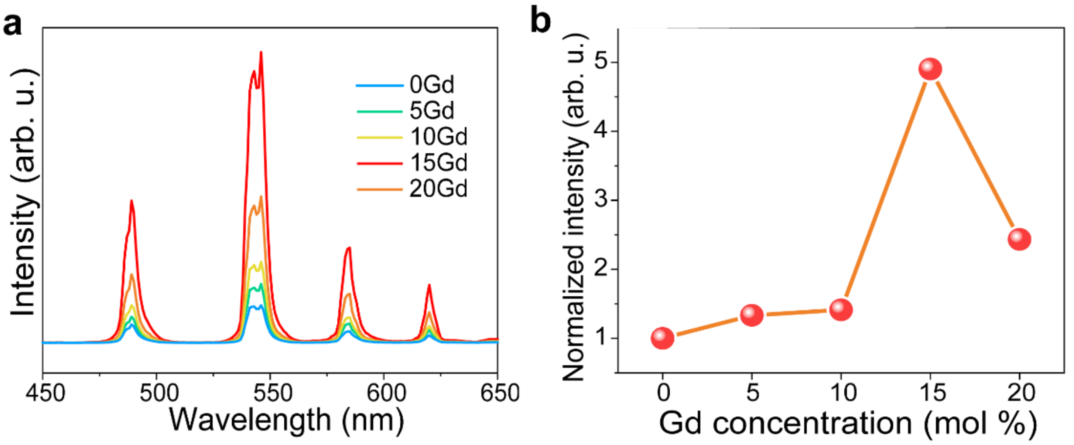

Supplementary Fig. 9 XEOL (**a**) and the corresponding normalized integral intensity variations **(b)** of the $NaLuF_4$: Gd/Tb NPs with different Gd^{3+} doping concentrations (0, 5, 10, 15, and 20 mol%). The case of 0 mol% is normalized to 1. Source data are provided as a Source Data file.

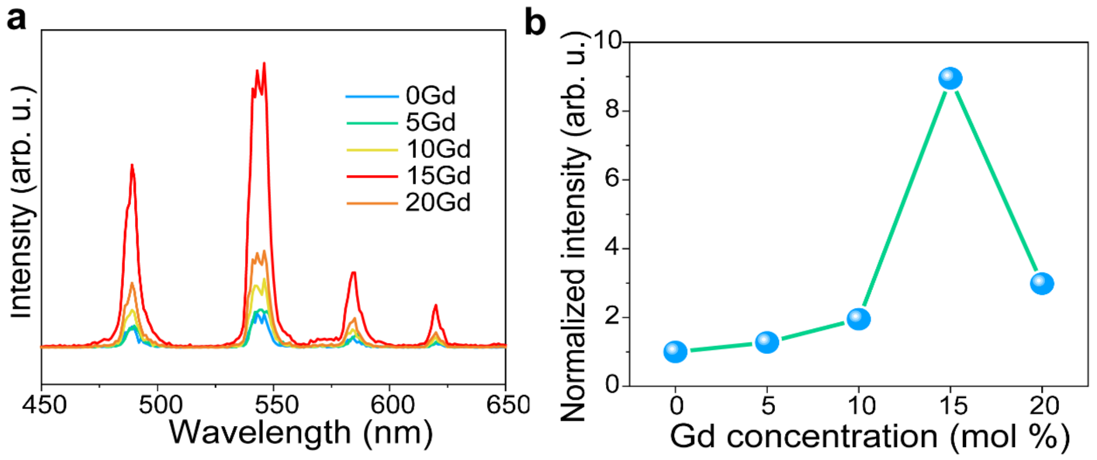

Supplementary Fig. 10 XEA (**a**) and the corresponding normalized integral intensity variations **(b)** of the $NaLuF_4$: Gd/Tb NPs with different Gd^{3+} doping concentrations (0, 5, 10, 15, and 20 mol%). The case of 0 mol% is normalized to 1. Source data are provided as a Source Data file.

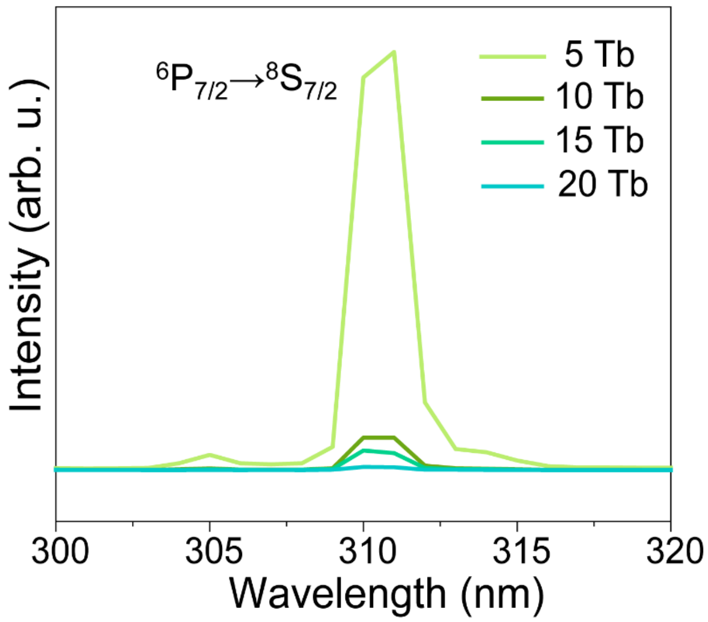

Supplementary Fig. 11 XEOL spectra of the $NaLuF_4$: 15Gd/Tb NPs with different Tb^{3+} doping concentrations (5, 10, 15, and 20 mol%). Source data are provided as a Source Data file.

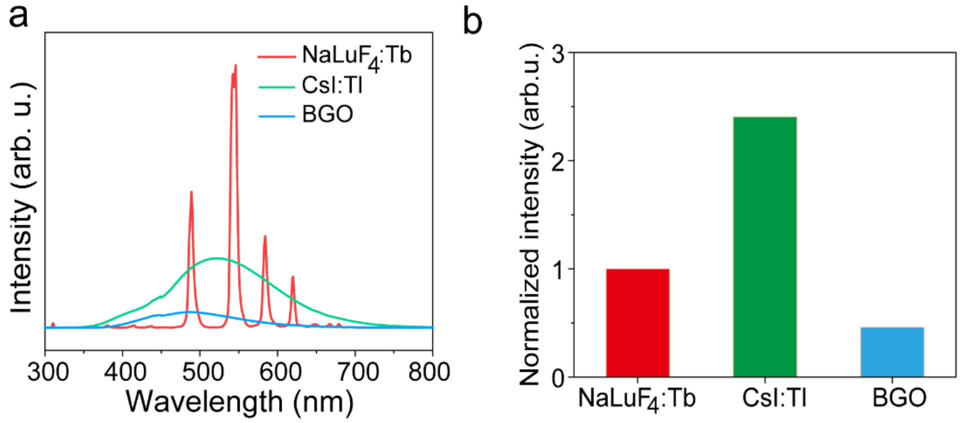

Supplementary Fig. 12 Compared XEOL spectra (**a**) and the corresponding normalized integral intensities (**b**) of the $NaLuF_4$:Tb NPs, commercial CsI:Tl and BGO scintillators. Source data are provided as a Source Data file.

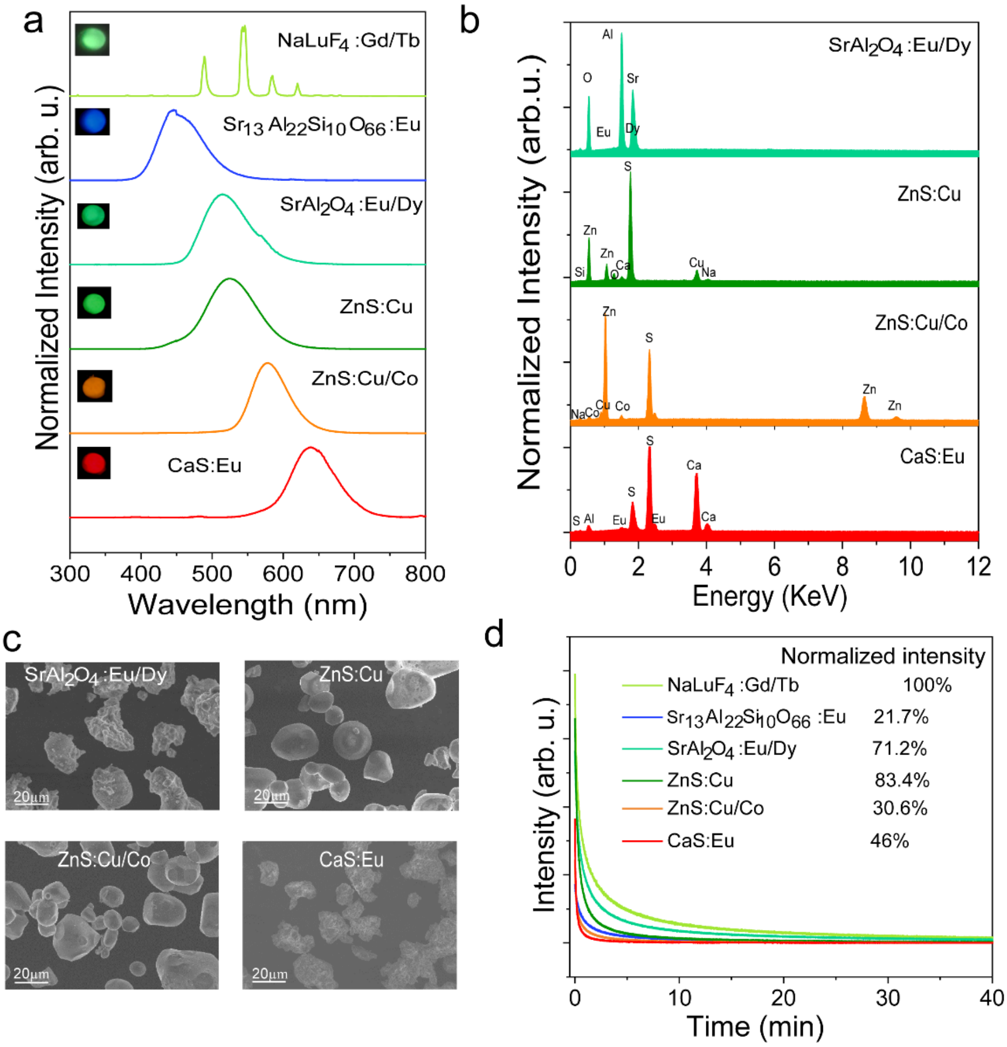

Supplementary Fig. 13 a Normalized XEOL spectra of the $NaLuF_4$: Tb NPs and various of conventional persistent phosphors. EDS spectrum (**b**) and SEM images (**c**) of different persistent phosphors. **d** XEA decay curves of those compared persistent phosphors. Inset shows their corresponding normalized initial XEA intensities. Source data are provided as a Source Data file.

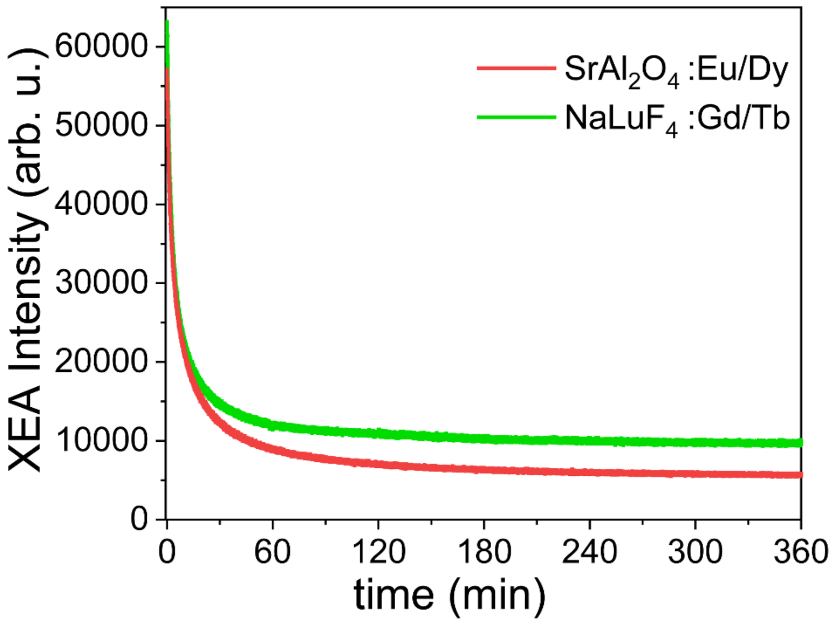

Supplementary Fig. 14 Compared XEA decay curves of the $NaLuF_4$: 15Gd/15Tb NPs and commercial $SrAl_2O_4$:Eu/Dy persistent phosphor (Edinburgh Instrument: FLS980 with R928 detector). Source data are provided as a Source Data file.

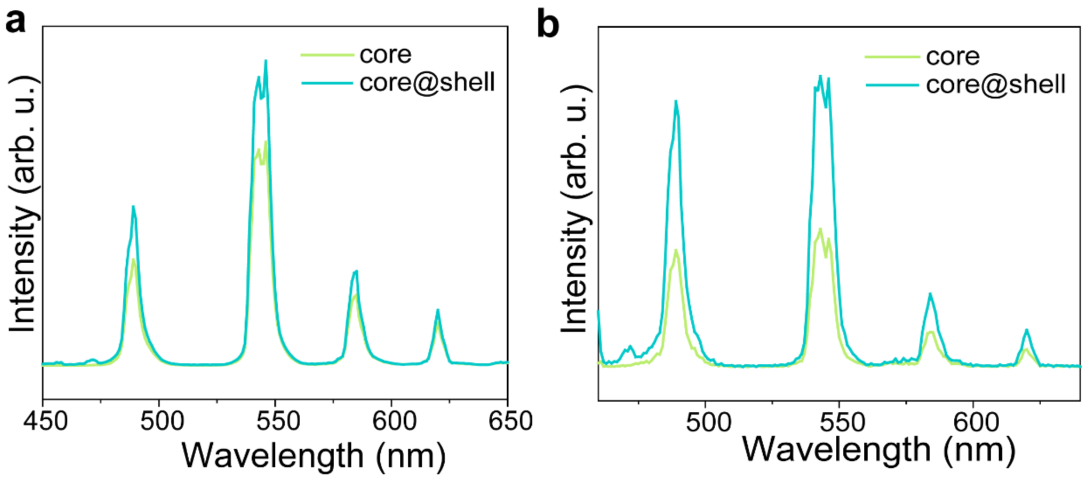

Supplementary Fig. 15 XEOL (**a**) and XEA **(b)** spectra of the $NaLuF_4$: Gd/Tb (15/15 mol%) core and $NaLuF_4$: Gd/Tb@$NaYF_4$ core@shell NPs. Source data are provided as a Source Data file.

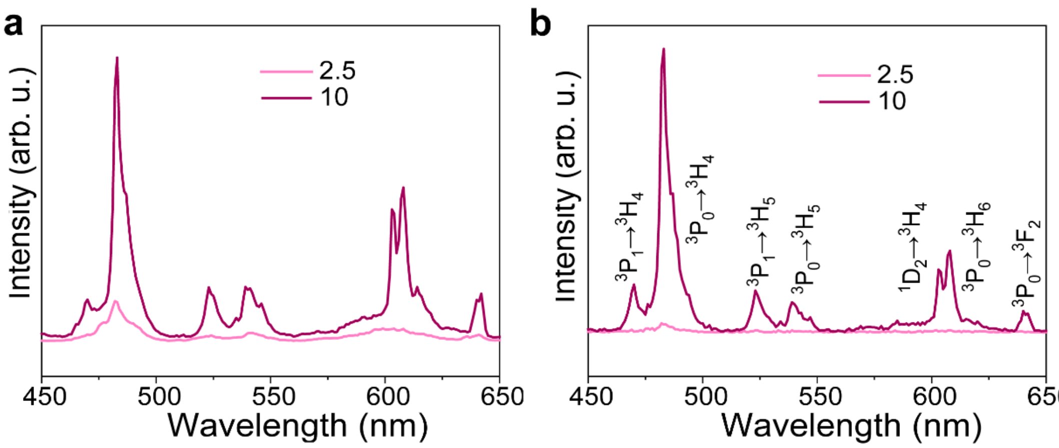

Supplementary Fig. 16 XEOL **(a)** and XEA **(b)** spectra of the $NaLuF_4$: Gd/Pr NPs prepared with [Na]/[RE] of 2.5 and 10. Source data are provided as a Source Data file.

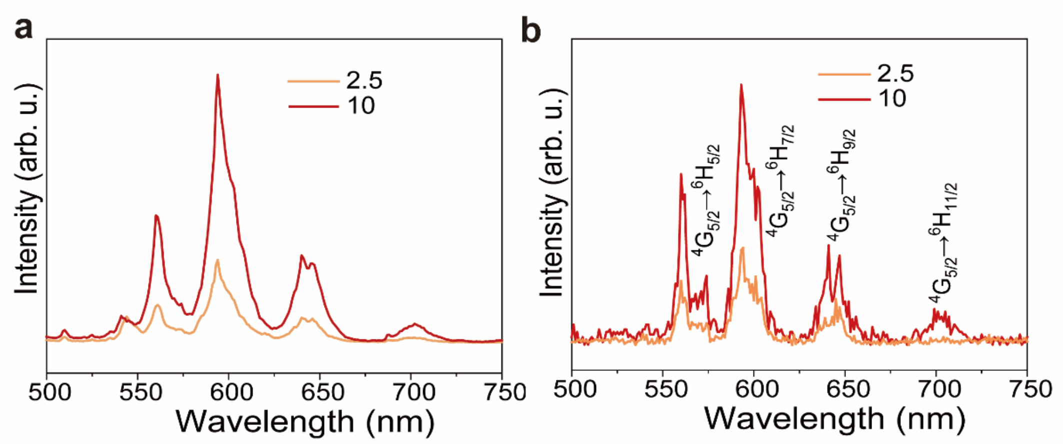

Supplementary Fig. 17 XEOL **(a)** and XEA **(b)** spectra of the $NaLuF_4$: Gd/Sm NPs prepared with [Na]/[RE] of 2.5 and 10. Source data are provided as a Source Data file.

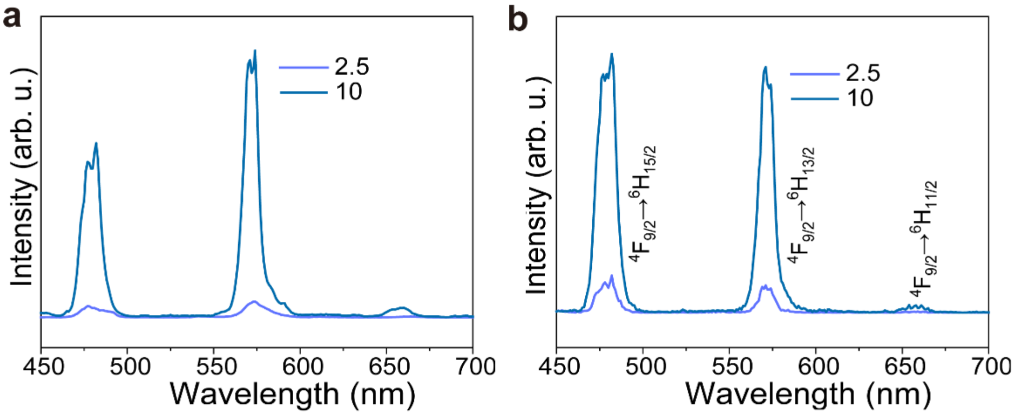

Supplementary Fig. 18 XEOL **(a)** and XEA **(b)** spectra of the $NaLuF_4$: Gd/Dy NPs prepared with [Na]/[RE] of 2.5 and 10. Source data are provided as a Source Data file.

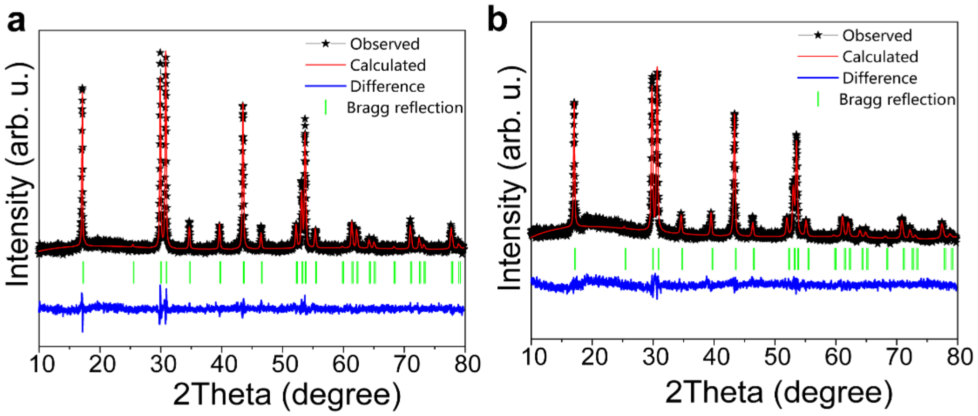

Supplementary Fig. 19 Rietveld refinement XRD patterns of the $NaLuF_4$: Gd/Tb NPs prepared with [Na]/[RE] of 2.5 **(a)** and 10 **(b)**. Source data are provided as a Source Data file.

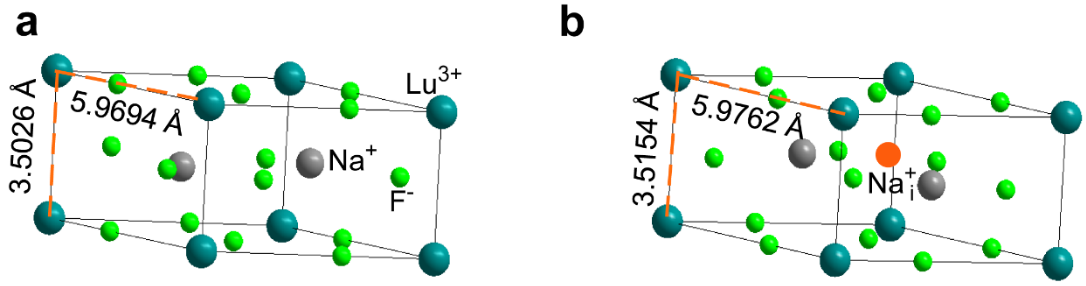

Supplementary Fig. 20 Schematic illustration of the hexagonal $NaLuF_4$ crystal structure with (**a**) and without (**b**) interstitial Na^+ (Na_i^+).

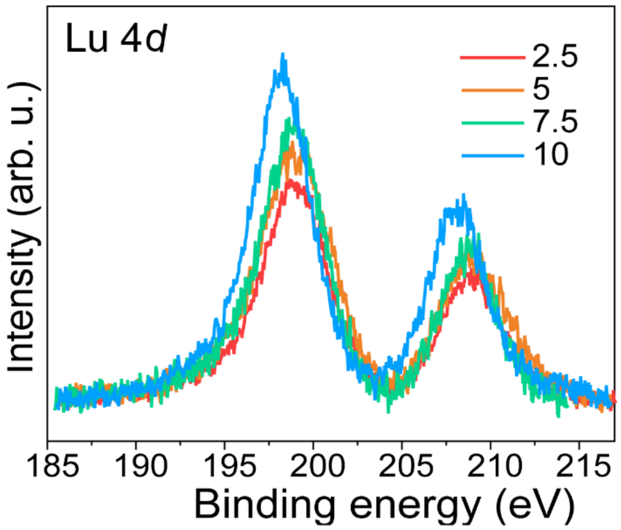

Supplementary Fig. 21 XPS spectra of Lu 4*d* for the $NaLuF_4$: Gd/Tb NPs prepared with different [Na]/[RE]. Source data are provided as a Source Data file.

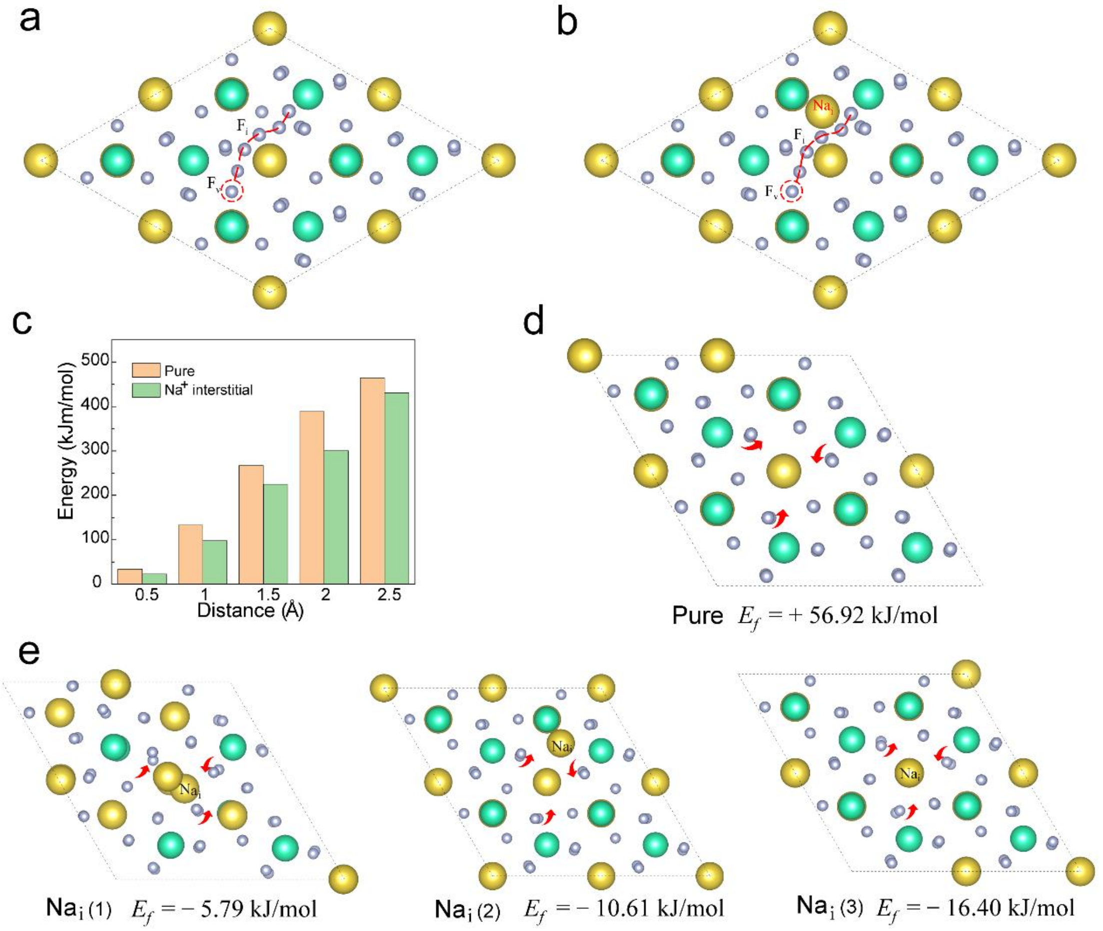

Supplementary Fig. 22 Schematic illustration of the Frenkel defects with different distances in the $NaLuF_4$ structure without (**a**) and with (**b**) intestinal Na^+ ions. **c** The corresponding calculated Frenkel defect formation energies. Formation of Frenkel defects in the $NaLuF_4$ structures without (**d**) and with (**e**) intestinal Na^+ ions at different sites under the consideration of the relaxation of all atoms. Source data are provided as a Source Data file.

Supplementary Discussion. The amount of X-ray induced Frenkel defects (n_F) can be expressed by the following equation[5]:

$$n_F = \sqrt{N_I N_i} e^{-\frac{E_f}{2kT}} \quad (1)$$

where N_I and N_i are the number of F^- lattices and interstitial sites, respectively, k and T are Boltzmann constant and temperature, respectively. In this occasion, the decrease of E_f can lead to an evident increase of n_F, which benefits the formation of high concentration traps.

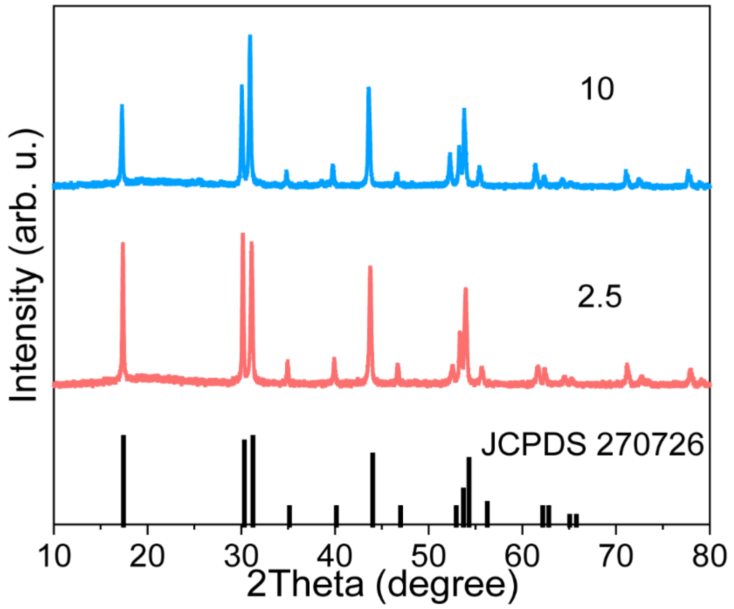

Supplementary Fig. 23 XRD patterns of the $NaYF_4$@$NaLuF_4$: Gd/Tb NPs inert-core@active-shell NPs prepared with [Na]/[RE] of 2.5 and 10 in the shell layer. The same batch of $NaYF_4$ core NPs were divided into two equal parts, which then were employed for the growth of $NaLuF_4$: Gd/Tb NPs active-shell layers with [Na]/[RE] of 2.5 and 10. Source data are provided as a Source Data file.

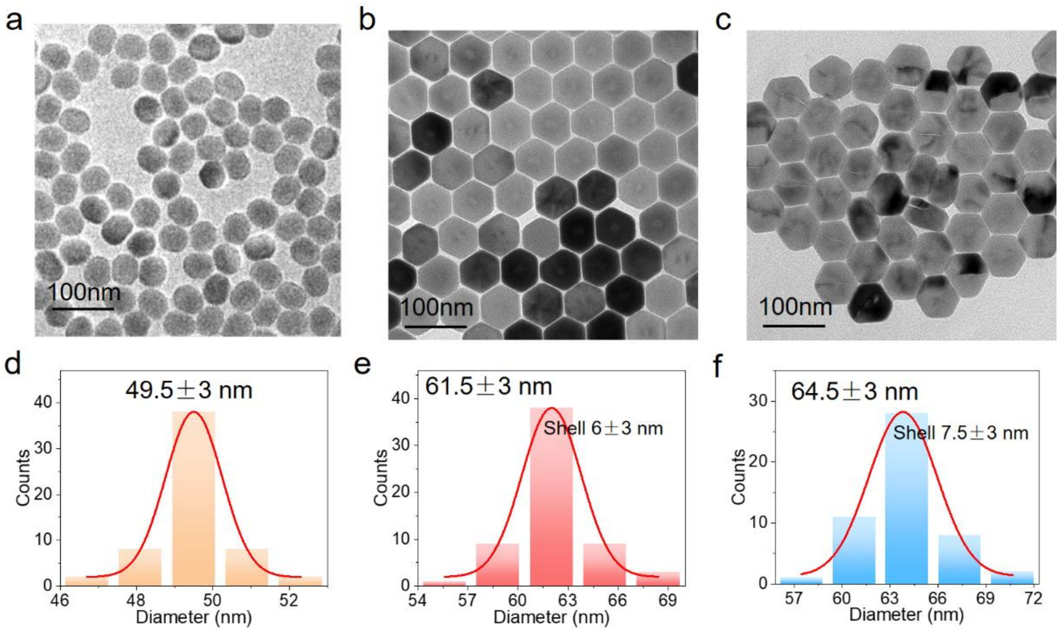

Supplementary Fig. 24 TEM images of the $NaYF_4$ core-only **(a)**, $NaYF_4$@$NaLuF_4$: Gd/Tb NPs inert-core@active-shell NPs prepared with [Na]/[RE] of 2.5 **(b)** and 10 **(c)** in the shell layer. **(d-f)** are their corresponding histogram size distributions of the top face. Source data are provided as a Source Data file.

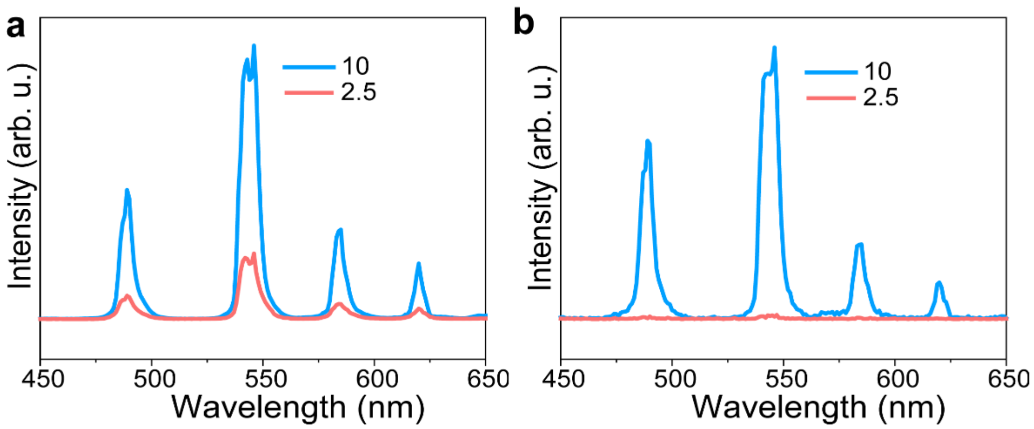

Supplementary Fig. 25 XEOL **(a)** and XEA **(b)** of the $NaYF_4$@$NaLuF_4$: Gd/Tb NPs inert-core@active-shell NPs prepared with [Na]/[RE] of 2.5 and 10 in the shell layer. Source data are provided as a Source Data file.

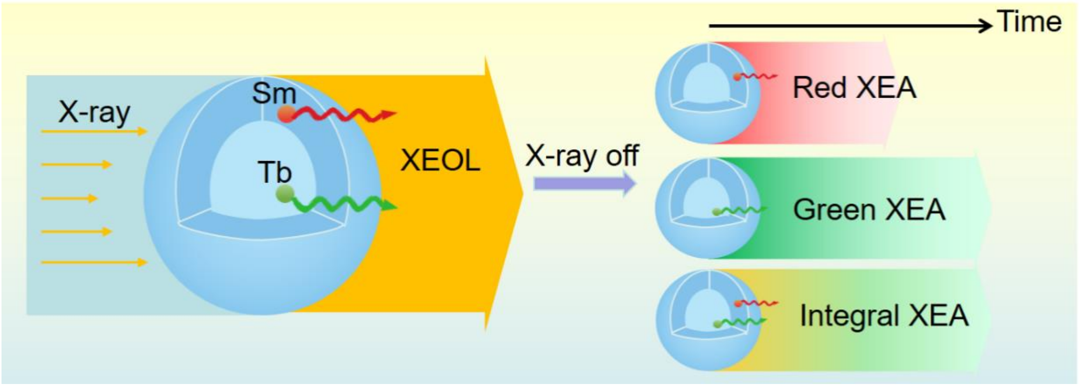

Supplementary Fig. 26 Schematic illustration for the time-dependent color evolution process in the $NaLuF_4$: Tb@$NaLuF_4$: Sm core@shell NPs.

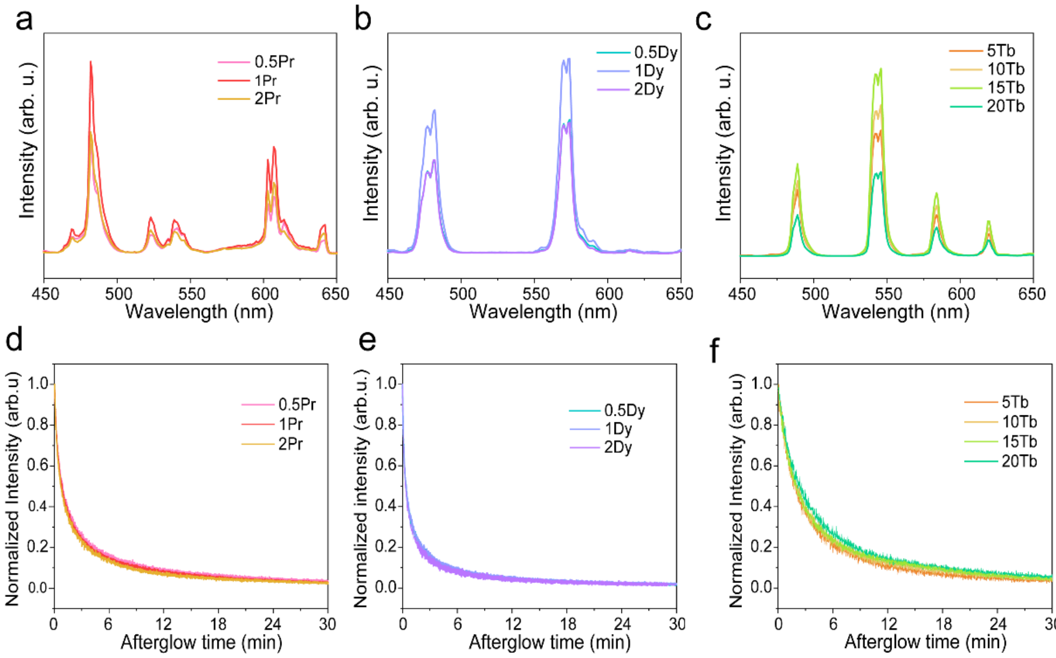

Supplementary Fig. 27 XEOL spectra and XEA decay curves of different lanthanide activators, **a, d** Pr (0.5, 1, 2 mol%), **b, e** Dy (0.5, 1, 2 mol%), **c, f** Tb (5, 10, 15, 20 mol%). Source data are provided as a Source Data file.

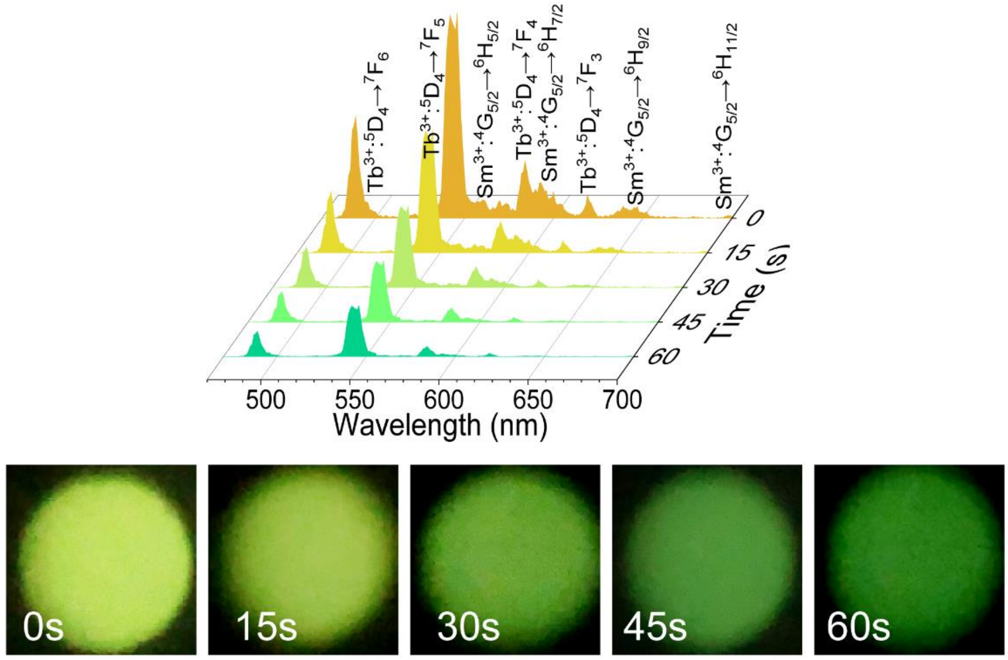

Supplementary Fig. 28 XEA spectra and corresponding photographs with different delay times of the Tb@Sm NPs. Source data are provided as a Source Data file.

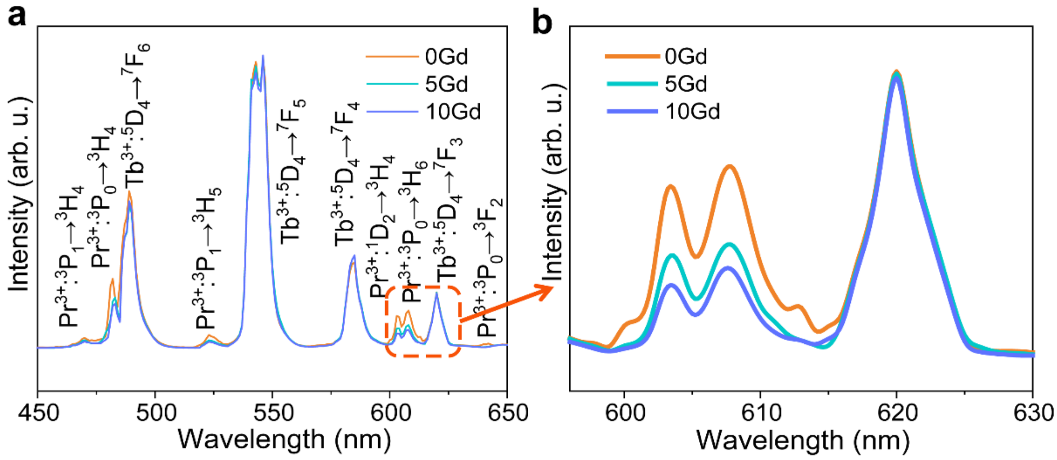

Supplementary Fig. 29 a XEOL of the NaLuF4: Gd/15Tb@NaLuF4: 15Gd/0.5Pr NPs with different Gd^{3+} ions concentrations in the core layer. **b** Zoom-in XEOL between 580-630 nm. Source data are provided as a Source Data file.

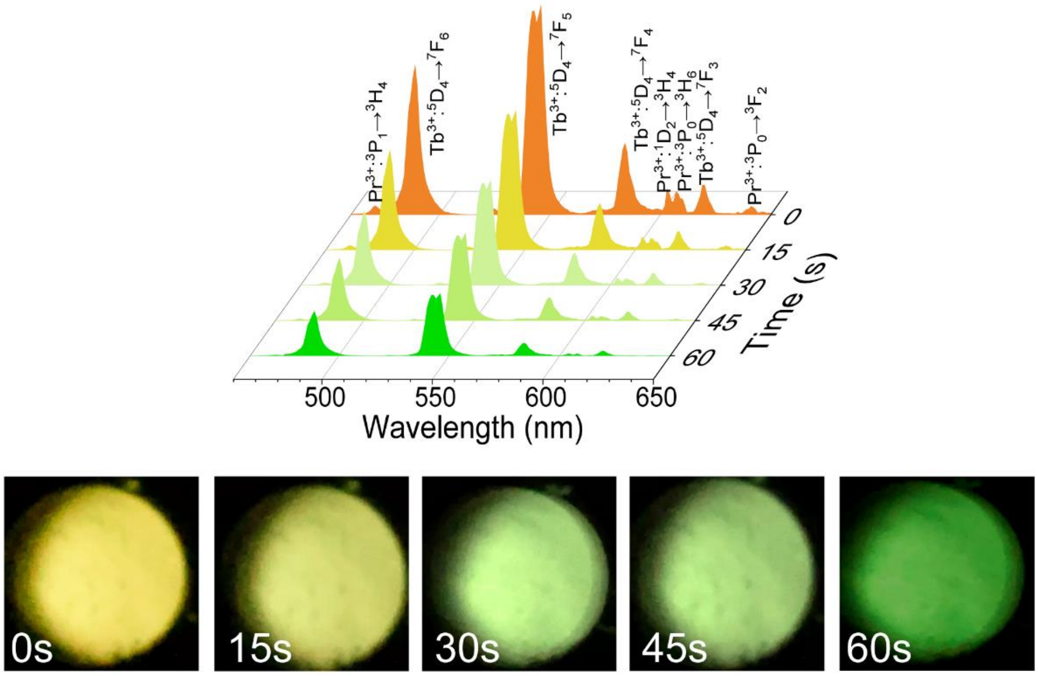

Supplementary Fig. 30 XEA spectra and corresponding photographs with different delay times of the Tb@Pr NPs. Source data are provided as a Source Data file.

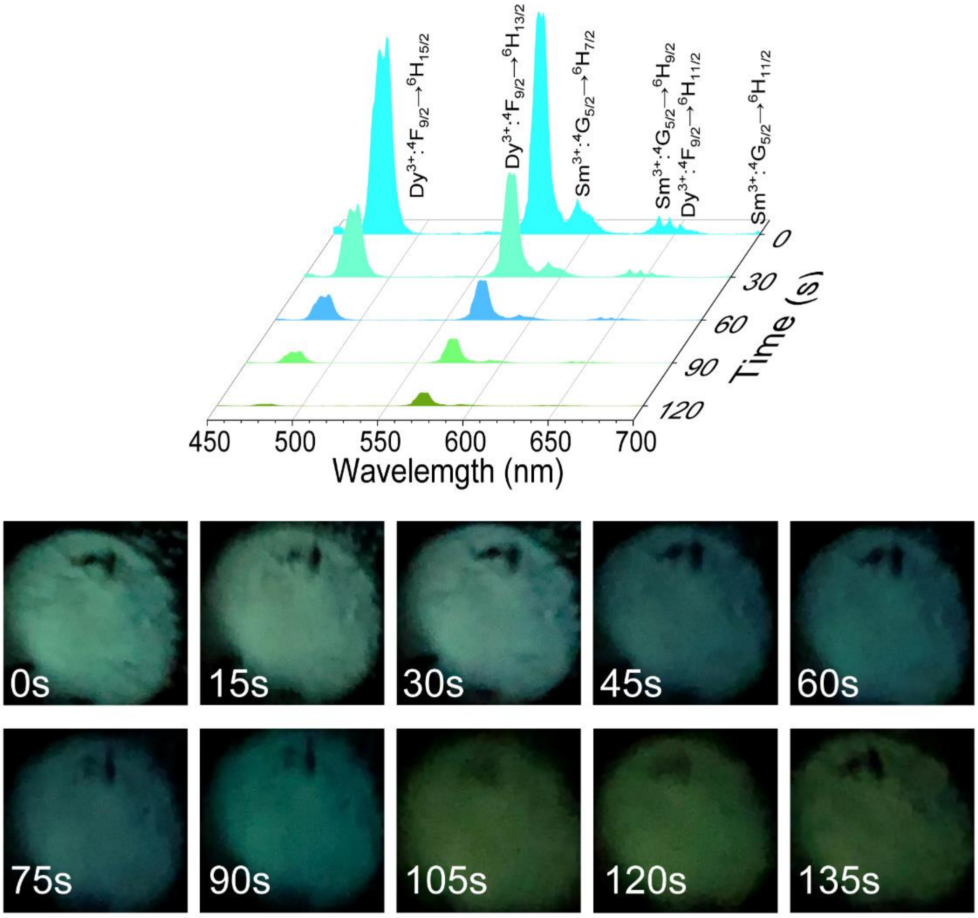

Supplementary Fig. 31 XEA spectra and corresponding photographs with different delay times of the Sm@Dy NPs. Source data are provided as a Source Data file.

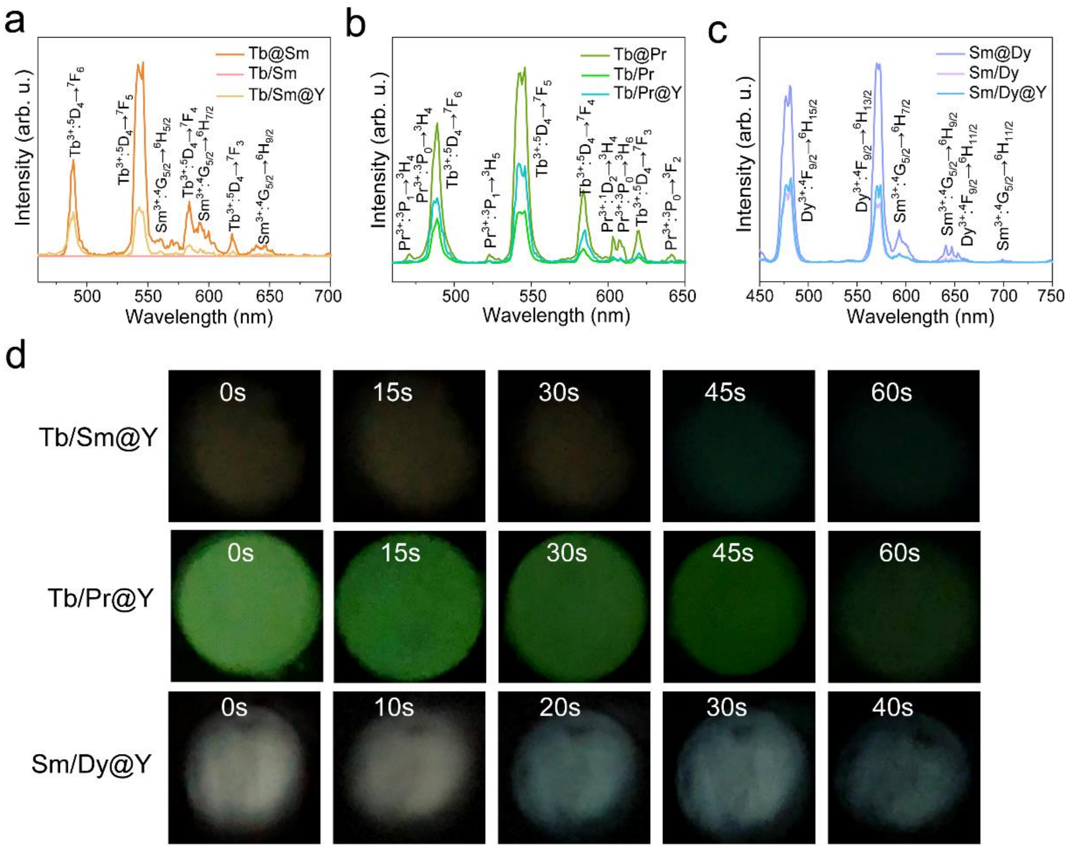

Supplementary Fig. 32 **a** XEA spectra of the $NaLuF_4$:15Gd/15Tb/0.5Sm (Tb/Sm), $NaLuF_4$:15Gd/15Tb/0.5Sm@$NaYF_4$ (Tb/Sm@Y) and $NaLuF_4$:15Gd/15Tb@ $NaLuF_4$:10Gd/0.5Sm (Tb@Sm) NPs. **b** XEA spectra of the $NaLuF_4$:15Gd/15Tb/0.5Pr (Tb/Pr), $NaLuF_4$:15Gd/15Tb/0.5Pr@$NaYF_4$ (Tb/Pr@Y) and $NaLuF_4$:15Tb@$NaLuF_4$:15Gd/0.5Pr (Tb@Pr). **c** XEA spectra of the $NaLuF_4$:15Gd/0.5Sm/0.5Dy (Sm/Dy), $NaLuF_4$:15Gd/0.5Sm/0.5Dy@$NaYF_4$ (Sm/Dy@Y) and $NaLuF_4$:15Gd/0.5Sm@$NaLuF_4$:15Gd/0.5Dy (Sm@Dy) NPs. **d** XEA photographs of the above NPs with different delay times. Source data are provided as a Source Data file.

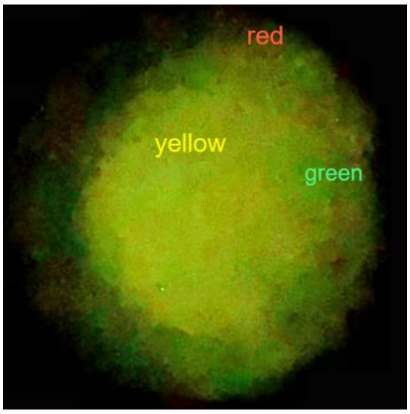

Supplementary Fig. 33 XEA photograph of the $NaLuF_4$:15Gd/15Tb and $NaLuF_4$:15Gd/0.5Sm mixture.

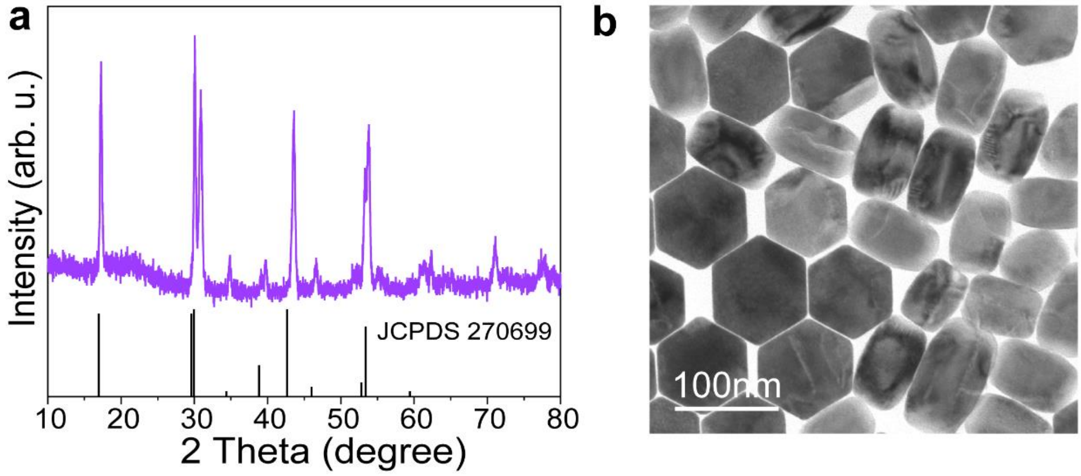

Supplementary Fig. 34 XRD pattern **(a)** and TEM image **(b)** of the $NaLuF_4$:15Gd/15Tb@$NaLuF_4$:15Gd/10Ce/0.5Sm@$NaGdF_4$:49Yb/1Tm core@shell@shell NPs. Source data are provided as a Source Data file.

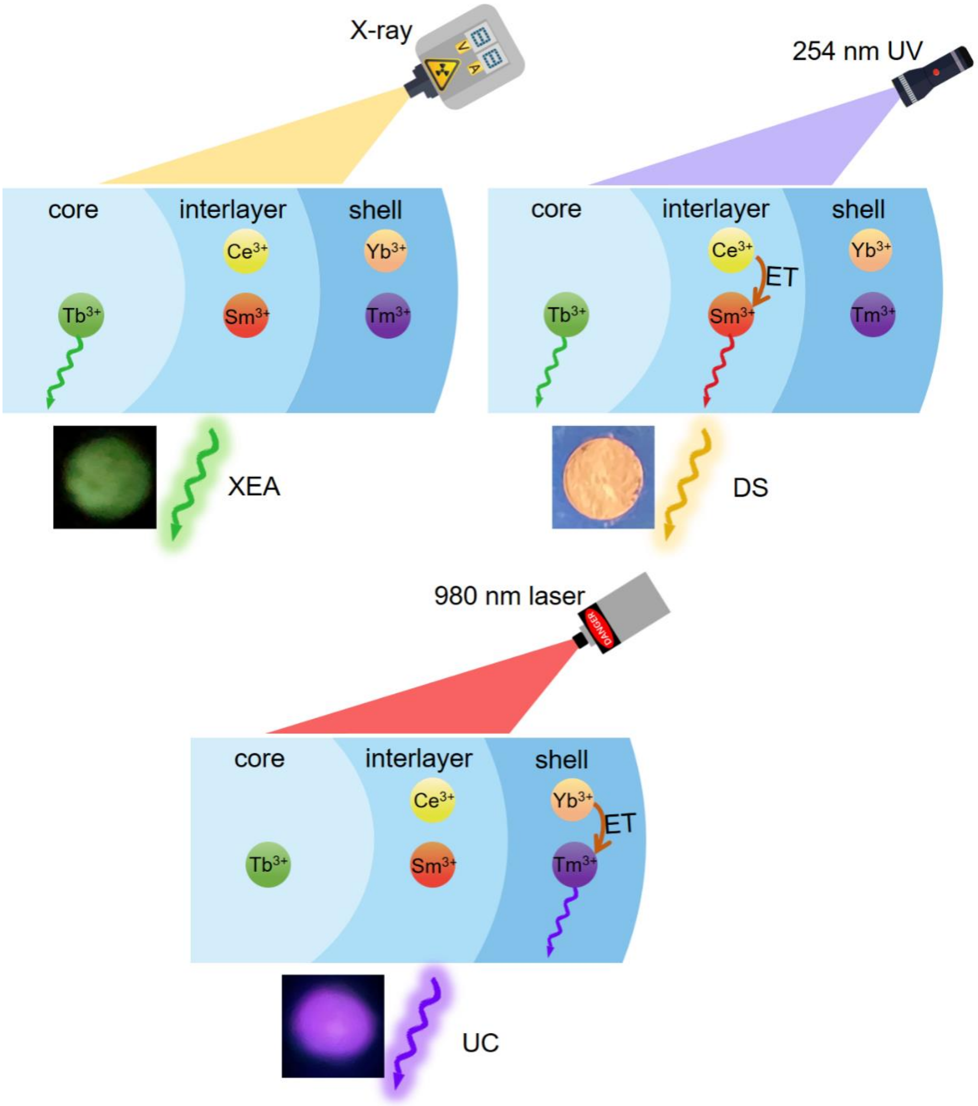

Supplementary Fig. 35 Schematic illustration for the excitation-dependent multicolour variations based on the core@shell@shell NPs.

Supplementary Discussion. Upon X-ray irradiation, the Tb^{3+} ions exhibited strong green afterglow. The XEA of Sm^{3+} ions is inhibited by decreasing the [Na]/[RE] to 2.5 and codoping Ce^{3+} ions. The input 254 nm UV photons were absorbed by Tb^{3+} and Ce^{3+} ions via the 4f-5d transition, which leads to both red Sm^{3+} emission through Ce^{3+}→Sm^{3+} or Ce^{3+}→Gd^{3+}→Sm^{3+} energy transfer and green Tb^{3+} emissions. Thus, under 254 nm UV illumination, the bright sulfur yellow color was observed. Under 980 nm laser excitation, the input photons are absorbed by Yb^{3+} sensitizers and then transferred to Tm^{3+} activators, resulting in the medium orchid UC.

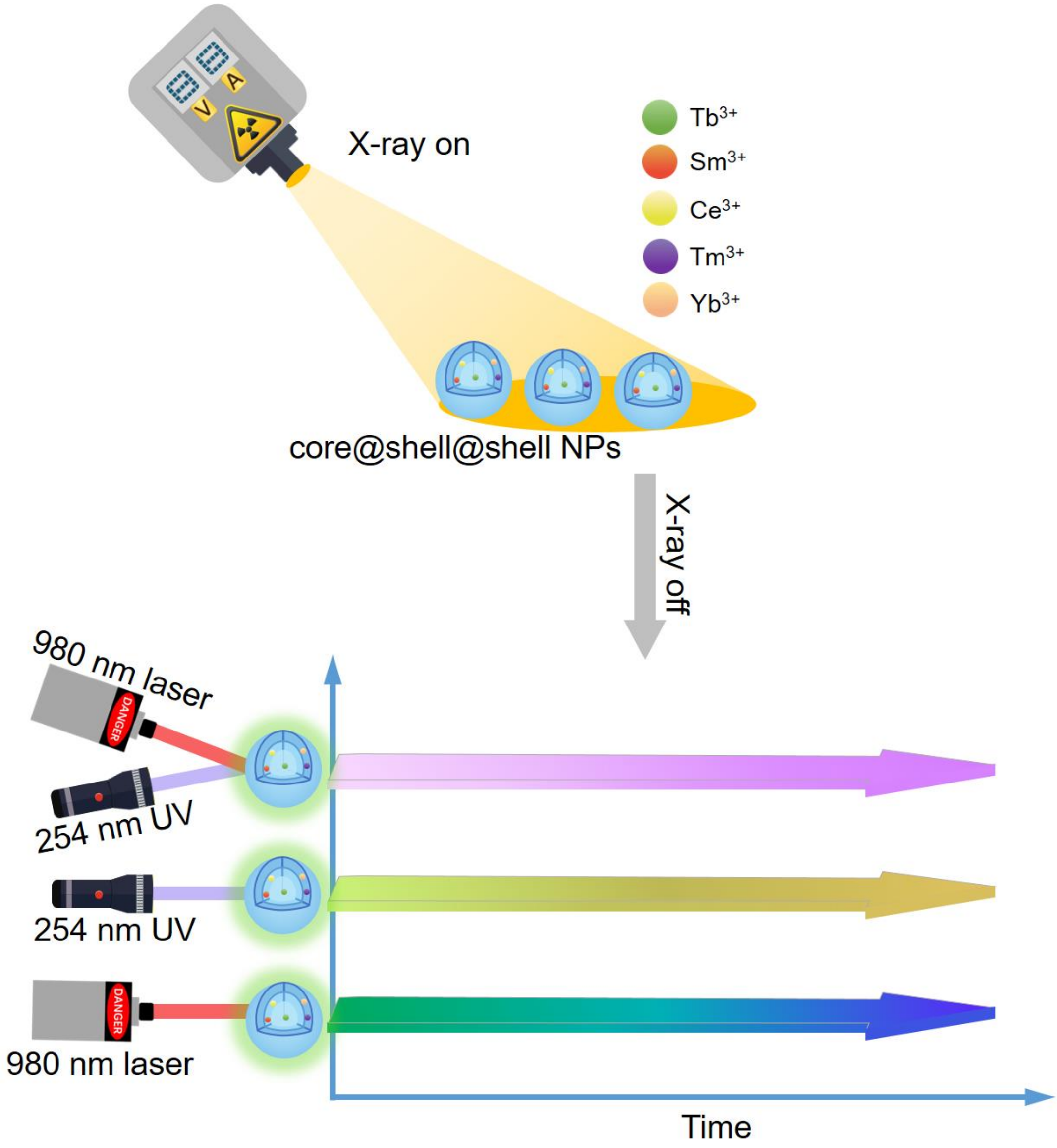

Supplementary Fig. 36 Schematic illustration for the manipulation of time-dependent multicolour evolution based on the core@shell@shell NPs.

Supplementary Discussion. The bowknot gel prepared by the $NaLuF_4$: Gd/Tb@$NaLuF_4$: Gd/Ce/Sm@$NaGdF_4$: Yb/Tm core@shell@shell NPs was irradiated by X-rays for 5 min. The XEA brightness was tuned by changing the irradiation power. Then the bowknot was excited under different excitation conditions. The XEA intensity is decreased over time, while the UC and DS intensity are not changed. As a result, the integral color was changed over time under different excitations. The initial color was controlled by tuning the pumping power.

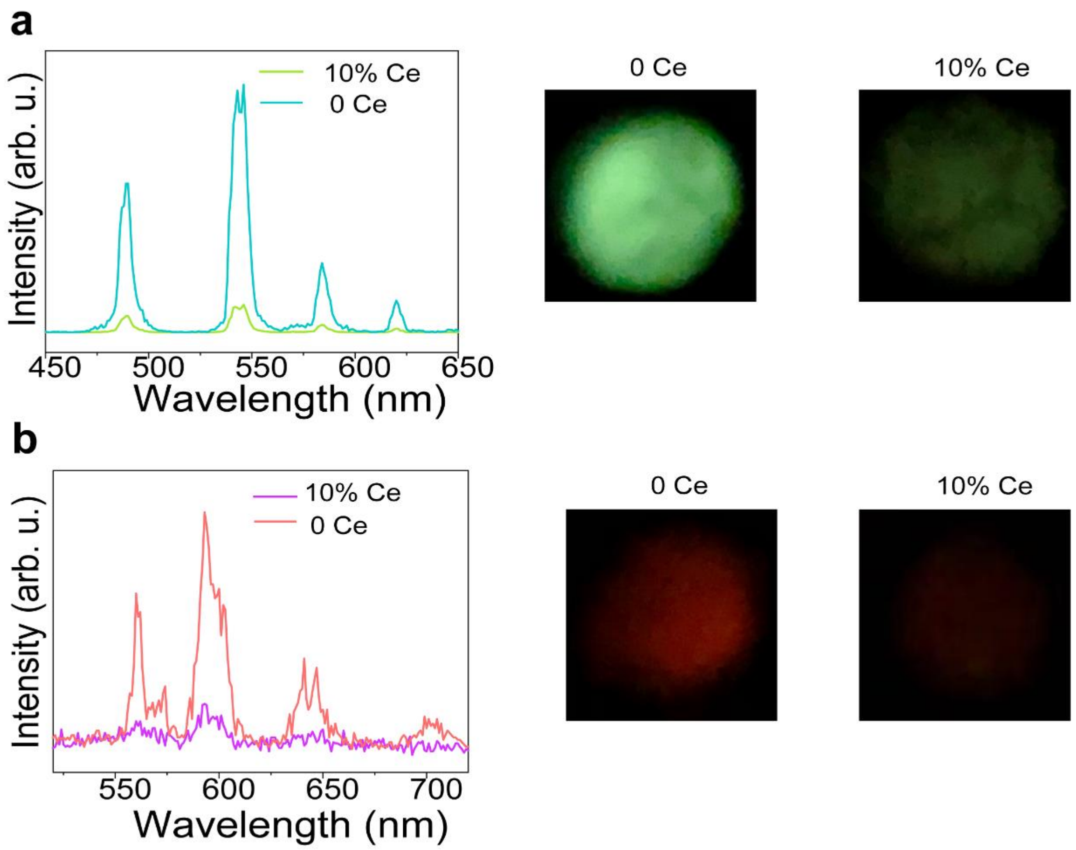

Supplementary Fig. 37 XEA spectra of the $NaLuF_4$: Gd/Tb (**a**) and $NaLuF_4$: Gd/Sm NPs (**b**) doped with and without Ce^{3+} ions (10 mol %) as well as their corresponding photographs. Source data are provided as a Source Data file.

Supplementary Discussion. The Ce^{3+}, Gd^{3+} and Sm^{3+} ions compete to capture electrons from the traps. Compared with the parity forbidden transitions within the f-manifold of lanthanides, the $4f^n$-$4f^{n-1}5d^1$ optical transitions are often characterized by high radiative emission probability because the f-d transition is electrical-dipole allowed. In our case, the Ce^{3+} ion exhibits much larger absorption cross-section via 4f-5d transition than those of Gd^{3+} and Sm^{3+} ions via 4f-4f transitions, thus, most of the electrons deposited in the traps will be captured by Ce^{3+} ions. Although the energy transfer processes from Ce^{3+} to Gd^{3+} and then to Sm^{3+} has been widely used to produce DS emission, it is hard to avoid energy loss during the energy transfer from Ce^{3+} to Gd^{3+} and from Gd^{3+} to Sm^{3+}. As a result, the incorporated Ce^{3+} ions will capture large number of electrons deposited in the traps, many of which are lost during the energy transfer processes.

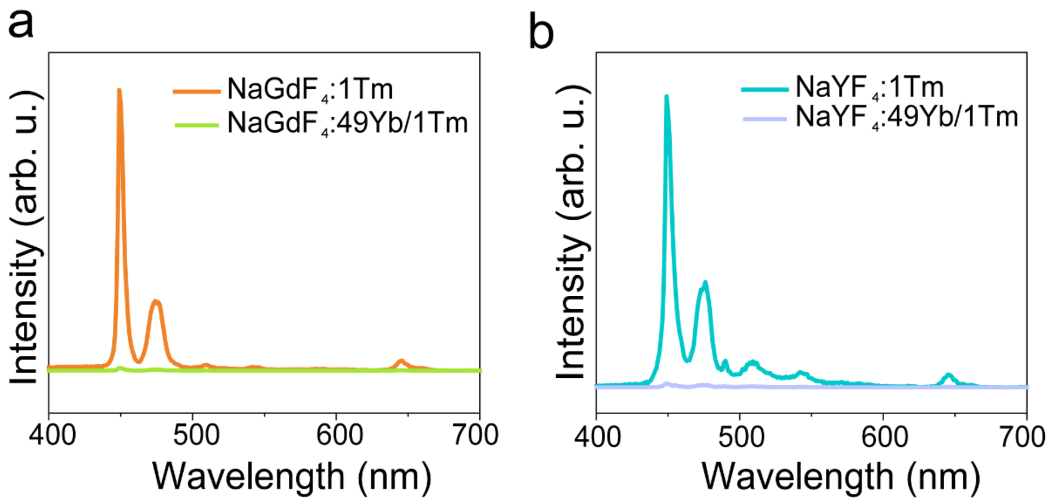

Supplementary Fig. 38 Compared XEA spectra of the $NaGdF_4$: 1Tm and $NaGdF_4$: 49Yb/1Tm (**a**), $NaYF_4$: 1Tm and $NaYF_4$: 49Yb/1Tm NPs (**b**). Source data are provided as a Source Data file.

Supplementary Discussion. The high concentration Yb^{3+} captures many electrons from the traps, and the efficiency of energy transfer from Yb^{3+} to Tm^{3+} is low for XEA process. Thus, the electron population in the Tm^{3+} is greatly reduced after the incorporation of Yb^{3+} ions.

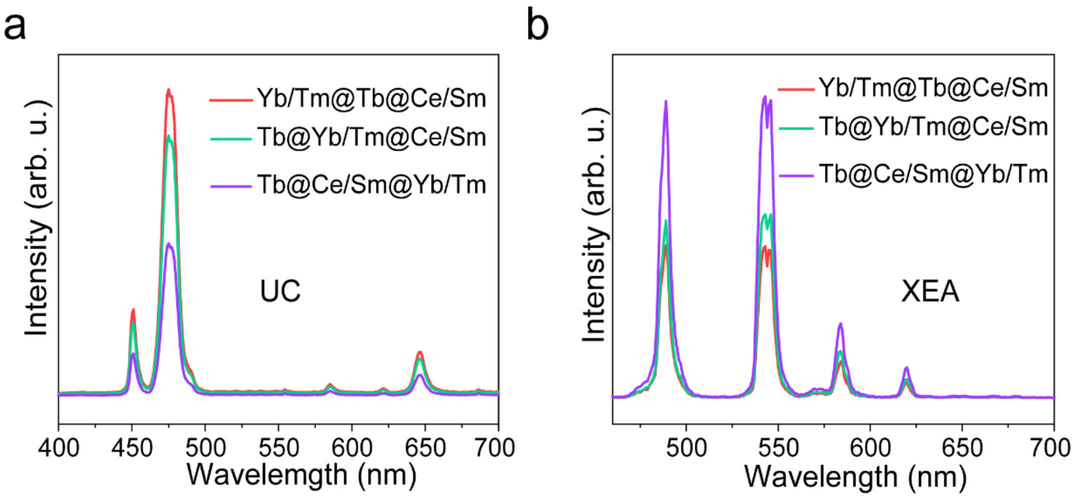

Supplementary Fig. 39 UC (**a**) and XEA (**b**) spectra of the Yb/Tm@Tb@Ce/Sm, Tb@Yb/Tm@Ce/Sm and Tb@Ce/Sm@Yb/Tm NPs. Source data are provided as a Source Data file.

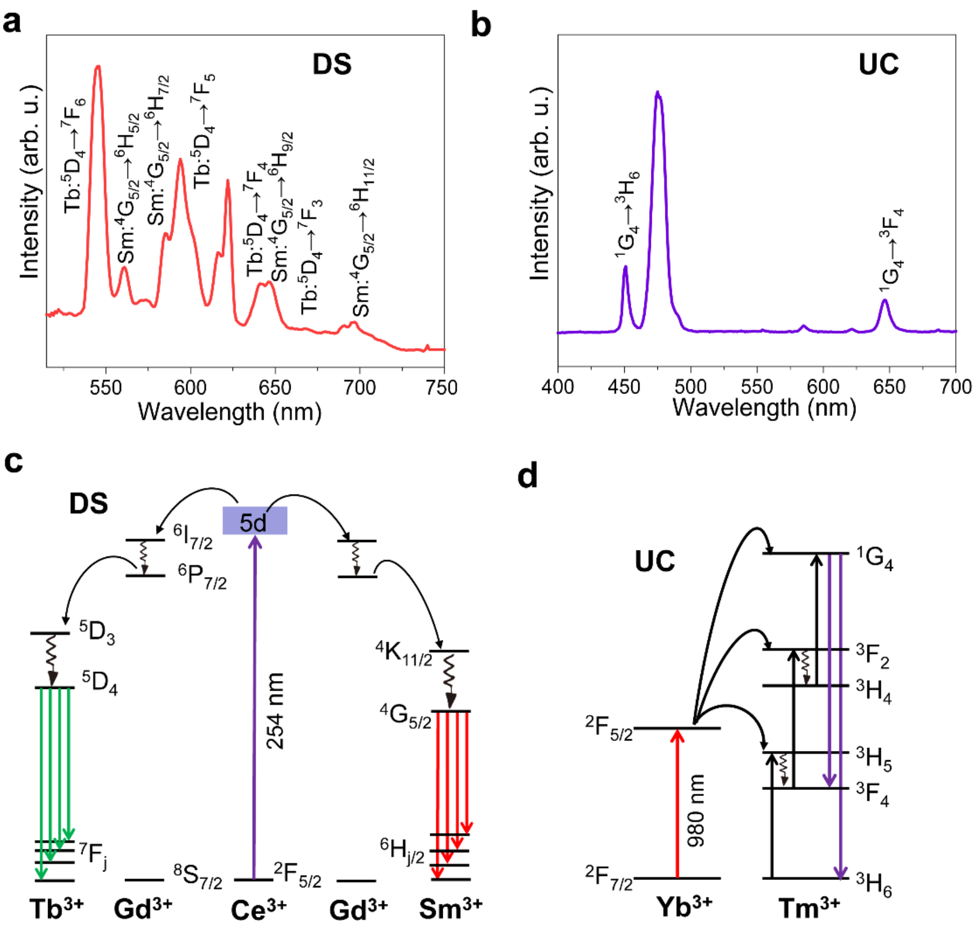

Supplementary Fig. 40 DS (**a**) and UC (**b**) emission spectra of the Tb@Ce/Sm@Yb/Tm core@shell@shell NPs. Proposed energy transfer processes of the DS (**c**) and UC (**d**). Source data are provided as a Source Data file.

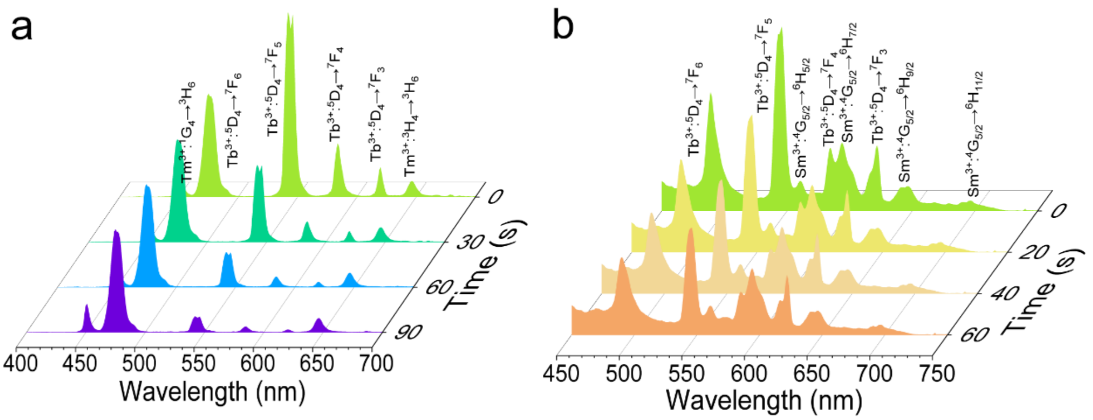

Supplementary Fig. 41 Time-dependent UC (a) and DS (b) spectra of the pre-X-ray-irradiated bowknot gel. Source data are provided as a Source Data file.

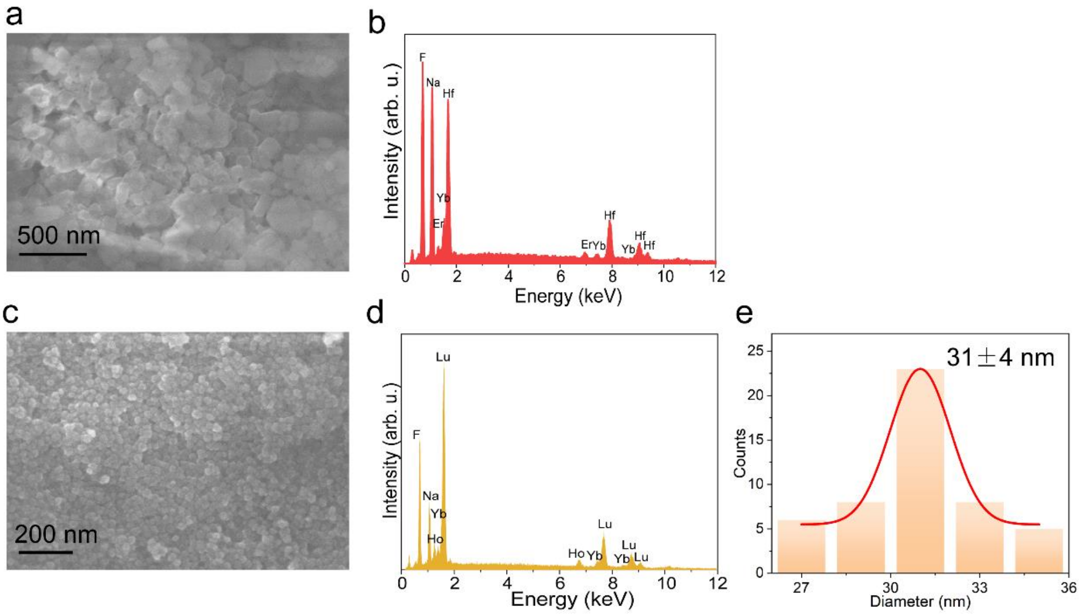

Supplementary Fig. 42 SEM image (**a**) and EDS spectrum (**b**) of the Na_3HfF_7:Yb/Er. SEM image (**c**), EDS spectrum (**d**) and size distribution (**e**) of the $NaLuF_4$:Yb/Ho. Source data are provided as a Source Data file.
